# Endogenous Biosynthesis of S-Nitrosoglutathione From Nitro-Fatty Acids in Plants

**DOI:** 10.3389/fpls.2020.00962

**Published:** 2020-06-30

**Authors:** Capilla Mata-Pérez, María N. Padilla, Beatriz Sánchez-Calvo, Juan C. Begara-Morales, Raquel Valderrama, Mounira Chaki, Lorena Aranda-Caño, David Moreno-González, Antonio Molina-Díaz, Juan B. Barroso

**Affiliations:** ^1^ Group of Biochemistry and Cell Signalling in Nitric Oxide, Department of Experimental Biology, Center for Advanced Studies in Olive Grove and Olive Oils, Faculty of Experimental Sciences, University of Jaén, Jaén, Spain; ^2^ Analytical Chemistry Research Group, Department of Physical and Analytical Chemistry, University of Jaén, Jaén, Spain

**Keywords:** nitro-fatty acids, nitric oxide, *S*-nitrosoglutathione, *S*-nitrosothiols, NO-signaling, nitric oxide donor, *Arabidopsis*, alkenal reductase

## Abstract

Nitro-fatty acids (NO_2_-FAs) are novel molecules resulting from the interaction of unsaturated fatty acids and nitric oxide (NO) or NO-related molecules. In plants, it has recently been described that NO_2_-FAs trigger an antioxidant and a defence response against stressful situations. Among the properties of NO_2_-FAs highlight the ability to release NO therefore modulating specific protein targets through post-translational modifications (NO-PTMs). Thus, based on the capacity of NO_2_-FAs to act as physiological NO donors and using high-accuracy mass-spectrometric approaches, herein, we show that endogenous nitro-linolenic acid (NO_2_-Ln) can modulate *S*-nitrosoglutathione (GSNO) biosynthesis in *Arabidopsis*. The incubation of NO_2_-Ln with GSH was analyzed by LC-MS/MS and the *in vitro* synthesis of GSNO was noted. The *in vivo* confirmation of this behavior was carried out by incubating *Arabidopsis* plants with ^15^N-labeled NO_2_-Ln throughout the roots, and ^15^N-labeled GSNO (GS^15^NO) was detected in the leaves. With the aim to go in depth in the relation of NO_2_-FA and GSNO in plants, *Arabidopsis* alkenal reductase mutants (*aer* mutants) which modulate NO_2_-FAs levels were used. Our results constitute the first evidence of the modulation of a key NO biological reservoir in plants (GSNO) by these novel NO_2_-FAs, increasing knowledge about *S*-nitrosothiols and GSNO-signaling pathways in plants.

## Introduction

Nitric oxide (NO), a small, gaseous, and highly reactive molecule able to cross cell membranes, has been described as an important biological messenger both in animal and plant systems (Stamler et al., 1992; Yu et al., 2014). In the last few years, diverse studies have described NO as a regulator involved in disease resistance, the response to different abiotic stresses, and as a key molecule in plant physiological processes such as stomatal closure, seed germination, iron homeostasis or several developmental processes (Delledonne et al., 1998; Garcia-Mata et al., 2003; Valderrama et al., 2007; Chaki et al., 2009; Begara-Morales et al., 2013).

NO and NO-related molecules such as nitrogen dioxide (NO_2_) or peroxynitrite (ONOO^−^) can interact with biomolecules, resulting in several changes such as the nitration of fatty acids, proteins, and nucleic acids or the *S*-nitrosation of proteins. Among these modifications, protein tyrosine nitration and *S*-nitrosation have been widely studied in animal and plant systems (Bartesaghi et al., 2007; Abello et al., 2009; Foster et al., 2009; Astier et al., 2012; Radi, 2012). Nevertheless, in the last few years a growing body of studies have highlighted the relevance of fatty acid nitration in living systems (Schopfer et al., 2011; Mata-Pérez et al., 2017). In this regard, nitro-fatty acids (NO_2_-FAs) result from the interaction of NO and NO-derived species with unsaturated fatty acids (Freeman et al., 2008). These molecules possess important biological properties, including the ability to release NO (Schopfer et al., 2005; Gorczynski et al., 2007; Mata-Pérez et al., 2016a) and the capacity to modify protein targets by a process specifically called nitroalkylation (Villacorta et al., 2007; Ichikawa et al., 2008; Bonacci et al., 2011; Kansanen et al., 2011; Geisler and Rudolph, 2012). However, the specific mechanism by which NO_2_-FAs are able to release NO in aqueous solutions remains unknown to date although different ways have been proposed. In this sense, based on a modified Nef-reaction, the generation of a hydroxy-nitroso intermediate capable of producing NO has been postulated (Schopfer et al., 2005; Baker et al., 2009). Furthermore, a rearrangement in the structure of NO_2_-FAs with the putative release of this gaseous molecule has also been noted (Lima et al., 2005; Gorczynski et al., 2007). On the other hand, NO_2_-FAs are also called nitroalkenes given the ability of the adjacent carbon to the nitro (NO_2_) group to act as a potent electrophile (Geisler and Rudolph, 2012). This carbon confers to NO_2_-FAs the capacity to be a potential target of nucleophilic molecules such as the thiol groups of proteins with the subsequent modulation of conformation, location, and activity of these protein targets. Through these modifications, NO_2_-FAs such as nitro-oleic (NO_2_-OA) or nitro-linoleic acids (NO_2_-LA) are able to promote vasodilator, antioxidant and anti-inflammatory effects in animal systems (Faine et al., 2010; Ambrozova et al., 2016; Mata-Pérez et al., 2016b; Kansanen et al., 2017; Turell et al., 2017; Verescakova et al., 2017). Recently, the endogenous occurrence of NO_2_-Ln has been also described in several plant species such as *Arabidopsis thaliana*, *Pisum sativum*, and *Oryza sativa* (Mata-Pérez et al., 2016b; Mata-Pérez et al., 2017). Moreover, this NO_2_-FA is capable of launching a defence response through the induction of different heat-shock proteins (HSPs) and several antioxidant enzymes (Mata-Pérez et al., 2015; Mata-Pérez et al., 2016b) and hence the outstanding relevance of these signaling molecules both in animal and plant systems. In this transcriptomic analysis, the 2-alkenal reductase (AtAER, AT5G16970) was identified to be up-regulated by NO_2_-Ln. This AtAER belongs to a NADPH-dependent reductases family that are involved in the detoxification of reactive carbonyls in plants (Mano et al., 2005; Yamauchi et al., 2011). In addition, AtAER appears to be phylogenetically related to animal’s prostaglandin reductase-1 (PGR-1) that is also an alkenal one/reductase (AOR) with the capacity to reduce the double bond of α,β unsaturated 2-alkenals (Yamauchi et al., 2011; Vitturi et al., 2013; Mesa et al., 2015). Interestingly, this PGR-1 has also been described as a nitroalkene reductase enzyme that is able to reduce the double bond from α,β-unsaturated alkenes and therefore catalyze the conversion of the electrophilic nitroalkenes to the non-electrophilic nitroalkane (Vitturi et al., 2013). Consequently, PGR-1 regulates cellular levels of NO_2_-FAs and mediates nitroalkene-related signaling pathways. In this line, AtAER, as the plant homologous of PGR-1, also regulates the cellular level of NO_2_-FAs in plants.

Most NO signaling functions are transmitted by their ability to modify the cysteine residues of the target proteins. The resulting *S*-nitrosothiols (SNOs) can alter the function, location, conformation, and activities of proteins in numerous eukaryotic signaling pathways. The role of these molecules have been related to numerous processes including plant immune signaling (Feechan et al., 2005; Chaki et al., 2009; Spoel and Loake, 2011) or the implication in different adverse environmental conditions such as high light intensity, darkness, or salinity (Valderrama et al., 2007; Corpas et al., 2008; Corpas et al., 2016; Begara-Morales et al., 2018; Begara-Morales et al., 2019). For instance, a rise in the levels of SNO has been associated with greater susceptibility to pathogen infection (Feechan et al., 2005; Kneeshaw et al., 2014) and it has also been proposed as a new wound signal in sunflower seedlings subjected to mechanical wounding (Chaki et al., 2011a).

Among different SNOs, highlight *S*-nitrosoglutathione (GSNO) constituting the *S*-nitrosated derivative of glutathione (GSH), the major intracellular antioxidant in plants. GSNO has been considered a major mobile biological reservoir of NO bioactivity (Espunya et al., 2012) and an essential component of NO-dependent signal transduction. GSNO has been located in vascular tissues, collenchyma cells and epidermal cells, pointing to this molecule as a mobile NO signal throughout the plant (Barroso et al., 2006; Chaki et al., 2011a; Chaki et al., 2011b). In this sense, it bears mentioning that phloem has the notable ability to propagate messengers such as different reactive oxygen and nitrogen species (ROS and RNS) during plant defence (Gaupels et al., 2017) and that GSNO is involved as a key molecule in the systemic response to wounding stress (Espunya et al., 2012). Thus, GSNO is currently considered a NO carrier throughout the plant, thereby giving NO the capacity of a long-distance signaling molecule. On the other hand, the levels of this low-molecular-weight SNO are controlled by the enzyme GSNO reductase (GSNOR1), decomposing it to oxidized glutathione (GSSG) and hydroxylamine (Frungillo et al., 2014). In this scenario, knockout lines of this enzyme resulted in higher levels of GSNO and *S*-nitrosated proteins (the addition of a NO moiety to a thiol group into a specific subset of cysteine residues in proteins), indicating that GSNOR1 indirectly governs the level of protein *S*-nitrosation in plants (Feechan et al., 2005).

Based on the capability of NO_2_-FAs to release NO (Schopfer et al., 2005; Gorczynski et al., 2007; Mata-Pérez et al., 2016a) and the involvement of these molecules in key aspects of plant physiology, here we show that NO_2_-Ln is able to move through the plant and also has the capacity to modulate the levels of GSNO both *in vitro* and *in vivo*. Thus, data presented in this study provide novel information concerning the SNO biosynthesis mechanisms, indicating that modulation of cellular levels of NO_2_-FAs can directly influence the GSNO levels and, indirectly, SNOs. Thus, NO_2_-FAs can be considered key players regulating the NO-dependent signaling pathways, highlighting the relevance of understanding the metabolism of GSNO in plant systems.

## Materials and Methods

### Plant Material and Growth Conditions

*Arabidopsis* ecotype Columbia and *aer* mutant (SALK 005324C) plants were used in this study. The homocygosis of *aer* mutant (Alonso et al., 2003) was confirmed by PCR using the primers designed according to the Salk Institute Genomic Analysis Laboratory instructions **(** **Table S1** **)**. For the different analyses, 7-day-old and 45-day-old *Arabidopsis* (*Arabidopsis thaliana*) plants were used. Both wild-type (WT) and mutant seeds were surface-sterilized for 5 min in 70% (v/v) ethanol containing 0.1% (w/v) SDS, placed for 20 min in sterile water containing 20% (v/v) bleach and 0.1% (w/v) SDS, and washed four times in sterile water. Then, seeds were grown up to 7 days in 0.8% phytoagar Petri plates under controlled conditions. The 45-day-old *Arabidopsis* plants were obtained by sowing seeds in tubes with 1% phytoagar and growing them in a culture chamber for 7 days under anaerobic conditions. Afterward, seeds were transferred to hydroponic cultures with a specific growth medium (Cellier et al., 2004) and aeration in controlled conditions (Day: 16 h, 22°C. Night: 8 h, 18°C. Light intensity: of 100–120 µE m^−2^ s^−1^).

For treatments, ^15^NO_2_-Ln was firstly synthesized and quantified as previously described (Mata-Pérez et al., 2016b) for the synthesis of NO_2_-Ln but using ^15^NaNO_2_ (Sigma-Aldrich, 490814) as a nitrating agent. Because NO_2_-Ln is not commercially available, it was synthesized by a nitroselenation-oxidation-hydroselenoxide elimination sequence as previously described (Mata-Pérez et al., 2016b; Mata-Pérez et al., 2018) with minor modifications. Briefly, commercial linolenic acid (1.1 mmol) was incubated with solid mercury chloride (1.4mmol), phenylselenyl bromide (1.1 mmol) and ^15^NaNO_2_ (1.1 mmol) in a mixture of tetrahydrofuran-acentonitrile (1:1, v/v, 7.0 ml). This mixture was kept under Ar atmosphere for 4 h with continuous agitation. After removing solid suspension and solvent, the residue was dissolved in tetrahydrofuran (7.0 ml) and keep in a water-ice bath at 0°C. Then, a 30% hydrogen peroxide solution (11.0 mmol) was added dropwise and the mixture was maintained in the cooling bath for 20 min with continuous agitation. After allowing the sample to reach room temperature, the reaction crude was extracted with hexane (2 × 20 ml), washed with saturated aqueous sodium chloride, dry over anhydrous magnesium sulfate, filter and evaporate to dryness under reduced pressure. The residue was taken up in a hexane/ether/acetic acid mixture (5 ml, 80:20/1, v/v/v) and purified by flash column chromatography (silica gel 60, 230–400 mesh, Fluka, Buches, Switzerland) with a mixture of hexane/ether/acetic acid (80:20/1,v/v/v) and ensuring the purification of mononitrated linolenic acid. Finally, the fractions were analyzed by TLC, NMR and LC as described by Mata-Pérez et al. (2016b).

### Synthesis and Quantification of GSNO and GS^15^NO Standards

GS^14^NO and ^15^N-labeled GSNO (GS^15^NO) standards were prepared according to Hart (1985) by acid-catalyzed nitrosation of GSH (Sigma-Aldrich, G4251). Sodium nitrite (^14/15^N-labeled) (Sigma-Aldrich) was used to synthesize GS^14^NO/GS^15^NO, respectively. These compounds were quantified by measuring the absorbance at 334 nm (ϵ = 0.92 mM^−1^·cm^−1^).

### *In Vitro* Synthesis of GSNO From NO_2_-Ln and GSH

To study the formation of GSNO from NO_2_-Ln and GSH, we incubated several concentrations of NO_2_-Ln (0.1 and 1 mM) with 1 mM GSH in 50 mM phosphate buffer, pH 7.4, containing 0.1 mM DTPA (diethylenetetraminepentaacetic acid) for 1 h at RT^a^ with a gentle agitation. Reactions were conducted in darkness. Formation of GSNO was analyzed by LC-ES/MS (Bruker Esquire 6000, HPLC Agilent 1100) in negative ion mode. The different analytes were separated in a Waters Spherisorb ODS2 C18 column (3 mm × 125 mm, 5 μm). The mobile-phase composition was water (A) and acetonitrile (B) both with 1% of formic acid at a flow rate of 0.6 ml min^−1^. The gradient profile was as follows: 2–5% B (0–5 min); 40–95% B (6–22 min); and 95–2% B (22–25 min). MS/MS/MS (M3) analysis from GSNO was conducted in 0.40 V (335) and 0.60 V (305). The desolvation temperature was set at 400°C. In all cases, the data were collected, analyzed, and processed using Data Analysis Mass Spectrometry Software (Bruker, Daltonics).

### Detection of ^15^NO_2_-Ln in *Arabidopsis* Leaves and NO_2_-Ln in WT and *aer* Mutants Seedlings

For this experimental design, and using 45-day-old *Arabidopsis* plants, the nutrient solution was removed and the root system was gently washed with distilled water (Begara-Morales et al., 2014a; Begara-Morales et al., 2014b). These plants were then incubated with 1 mM ^15^NO_2_-Ln for 3 h, and this molecule was detected in leaves under non-stress conditions.

Lipid extracts from 45-day-old *Arabidopsis* leaves and 7-day-old WT and *aer* mutants seedlings were obtained using the Bligh and Dyer method (Bligh and Dyer, 1959) and prepared for LC-MS/MS detection of ^15^NO_2_-Ln and NO_2_-Ln, respectively, as it has been previously described (Mata-Pérez et al., 2016b; Mata-Pérez et al., 2018).

### Detection of Endogenous GSNO and GS^15^NO in *Arabidopsis*

For the endogenous detection of GSNO and GS^15^NO, the method used was similar to that described elsewhere (Tsikas et al., 2013) with some modifications. All steps were performed under cooling or 4°C and darkness. The samples were worked up fresh and analyzed quickly to avoid the degradation of the GSNO. In this regard, *Arabidopsis* leaves and seedlings were ground to a powder in a mortar with liquid nitrogen and suspended in an extraction buffer composed by 100 mM phosphate buffer, pH 7.8, containing 0.1 mM DTPA, 5 mM NEM (N-ethylmaleimide), 0.01 mM neucoproine (1/2, FW/V). Homogenates were centrifuged at 16,000*x*g for 10 min, 4°C and filtered using a standard 0.22-µm filter. Then, samples were ultra-filtered by centrifugation at 8,000*x*g, 30 min, 4°C using 5-kDa Vivaspin 2 Hydrosart 2-ml cartridges. The ultra-filtered sample was placed into the precooled (4°C) autosampler and 25 µl were injected into LC-MS/MS instrument.

GSNO endogenous content was quantified by carrying out an internal standard calibration with GSNO and GS^15^NO. Next, GSNO or GS^15^NO were added to aliquots of the sample in a range of 0–6 nM just before being analyzed by LC-MS/MS. Because GSNO is very unstable, the loss of this molecule during sample processing was evaluated by spiking 75 nM GS^15^NO into the extraction buffer. This loss during sample work-up was estimated at about 80% from the initial spiked amount.

The analytes were separated using a Dionex Ultimate 3000 rapid separation liquid chromatograph (RSLC) (Thermo Scientific, USA) instrument. This was equipped with an Agilent Zorbax Rapid Resolution High-Definition (RRHD) Eclipse Plus C18 column (2.1 mm × 100 mm, 1.8-µm particle size). The mobile-phase composition was water (A) and acetonitrile (B), both of them with 1% of formic acid at a flow rate of 0.6 ml min^−1^. The temperature of the column was 25°C and the injection volume was 20 µl. The gradient profile was as follows: 0 min, 0% B; 4 min, 100% B; 5 min, 100% B; 7 min, 0% B; 8 min, 0% B; 10 min.

The UHPLC system was connected to a TSQ Quantiva triple quadrupole (QqQ) (Thermo Scientific, USA) equipped with a heated electrospray ionization probe (HESI) operating in positive ion mode with the following operation parameters: spray voltage: 4,500 V; sheath gas 45; aux gas 5 arbitrary units; ion transfer tube temperature 150°C; vaporizer temperature 300°C; collision gas (CID), 1.5 mTorr. Multiple-reaction monitoring (MRM) transitions were optimized for each compound **(** **Table S2** **)**. XCalibur software 3.0.63 (Thermo Fisher Scientific, San José, CA, USA) was used for method development and data analysis.

### Quantitative Real-Time Reverse Transcriptase-PCR (qRT-PCR)

RNA isolation and gene expression of AER by qRT-PCR were performed in WT and *aer* 7-day-old *Arabidopsis* seedlings as previously described (Begara-Morales, 2014b RNA seq) using *Actin 12* (AT3G46520) as internal standard. The specific primers used are listed in **Table S1**.

### Crude Extracts of *Arabidopsis* Seedlings and Immunodetection of AER

Seven-day-old WT and *aer Arabidopsis* seedlings were ground to a powder in liquid nitrogen using a mortar and pestle, and the resulting powder was suspended in the extraction buffer (100 mM Tris-HCl buffer, pH 7.5, containing 0.1mM EDTA, 7% (w/v) PVPP, 5% Suc, 0.0005% Triton X-100, 1 mM PMSF, 15 mM DTT, and a commercial cocktail of protease inhibitors (AEBSF, 1,10-phenantroline, pepstatin A, leupeptine, bestatine, and E-64 from Sigma-Aldrich; 1/2, FW/v). Then, the crude extracts were centrifuged twice at 3,000*x*g for 6 min. Total protein content was analyzed by Bradford assay and separated by 10% SDS-PAGE and transferred to PVDF membranes (Immobilon P, Millipore, Bedford, MA, USA). For AER immunodetection, an specific antibody against *Arabidopsis* AER (Mano et al., 2005) was used at a dilution of 1:1000 and the immunoreactive band was detected using a photographic film (Hyperfilm, Amersham Pharmacia Biotech) with an enhanced chemiluminescence kit (ECL-PLUS, Amersham Pharmacia Biotech).

### Lipid Extraction and Fatty Acid Analysis

Lipid extracts from 7-day-old WT and *aer Arabidopsis* plants were obtained by the Bligh and Dyer method (Bligh and Dyer, 1959) and the content of fatty acids was analyzed by gas mass spectrometry (Agilent 7890A) as previously described (Mata-Pérez, 2015). Briefly, the lipid fractions were evaporated under a stream of nitrogen and dissolved in benzene and Meth-Prep II (Alltech Chemicals Cat. No. 18007) GC reagent to perform the transesterification of the lipid fractions. Following the derivatization stage, a GC/MS analysis was carried out by injecting a 1-μl solution. Analyses were carried out in a 7890A GC system (Agilent, USA) equipped with an SP-2560 capillary column (100 m × 0.25 mm × 0.25 μm) and a Quattro micro GC mass spectrometer (Waters, USA). The GC column procedure was as follows: initial temperature 140°C, maintained for 5 min, increased at 4°C min^−1^ to 250°C with a split ratio at injector port of 1:10. A standard oil mixture (Supelco ref. 18919-1AMP) was used to calibrate the gas chromatograph.

### Statistical Analysis

To estimate the statistical significance between means, the data were analyzed by Student’s t-test. The differences were significant at p < 0.05. For each series of experiments, at least three independent biological replicates have been performed with three technical replicates per biological assay.

## Results

### *In Vitro* Synthesis of GSNO From GSH and NO_2_-Ln

Based on the demonstrated ability of NO_2_-FAs acting as NO donors (Schopfer et al., 2005; Gorczynski et al., 2007; Mata-Pérez et al., 2016a), the *in vitro* generation of GSNO from the reaction between GSH and NO_2_-Ln was analyzed by LC-ES/MS in negative ion mode **(** **Figure S1** **)**.

**Figure 1:**
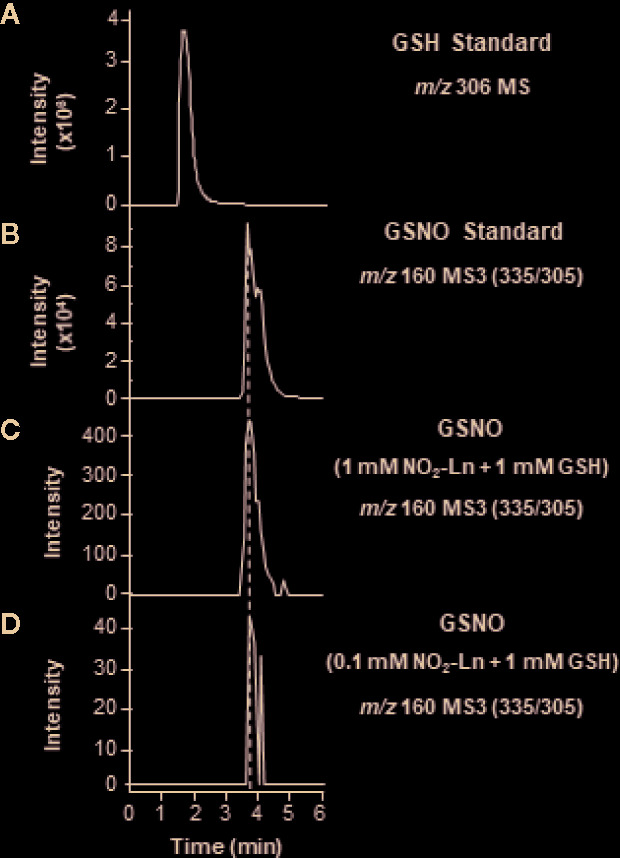
*In vitro* synthesis of GSNO from NO_2_-Ln and glutathione (GSH). For the *in vitro* generation of GSNO, 1 mM GSH was incubated with 1 and 0.1 mM NO_2_-Ln, as is described in *Materials and Methods*. **(A, B)** show GSH and GSNO standards, respectively. **(C, D)** display GSNO generated after the incubation of 1 mM GSH with 1 and 0.1 mM NO_2_-Ln, respectively. Peaks refer to total ion intensity. Vertical dashed lines indicate peaks with the same retention time. *m/z* is mass-to-charge ratio. MS indicates full ion mass spectra. MS3 is MS/MS/MS ion fragmentation.

In this sense, the full mass ion spectra (MS) of GSNO standard showed a major ion product with *m/z* of 335 corresponding to this low-molecular weight SNO when it was analyzed in negative ion mode **(** **Figure S1A** **)**. Then the MS/MS (MS2) spectra displayed a major fragment with *m/z* of 305 **(** **Figure S1B** **)** corresponding to the homolytic dissociation of the *S*-nitroso group for generating the protonated glutathionyl radical ([GS+H]^·+^) and the neutral radical ^·^NO (30 Da). The MS/MS/MS (MS3) fragmentation led to the detection of an ion fragment with *m/z* of 160 which confirmed GSNO occurrence **(** **Figure S1C** **)**. Afterward, different concentrations of NO_2_-Ln (0.1 and 1 mM) were incubated with 1 mM GSH and studied by LC-ES/MS3 **(** **Figure 1** **)**. Under these conditions, a chromatographic peak with MRM transition of 160 *m/z* was detected in both NO_2_-Ln analyzed concentrations **(** **Figures 1C, D** **)**. These peaks shared the same retention time as GSNO standard but not with GSH standard, thus confirming the formation of GSNO from the reaction between NO_2_-Ln and GSH **(** **Figures 1A, B** **)**. Additionally, product ions of the GSNO formed after reaction between NO_2_-Ln and GSH showed the same fragmentation pattern as synthetic GSNO **(** **Figure S2** **)**, and thereby confirmed the formation of GSNO.

### Mobilization of NO_2_-Ln Throughout *Arabidopsis* Plants

**Figure 2:**
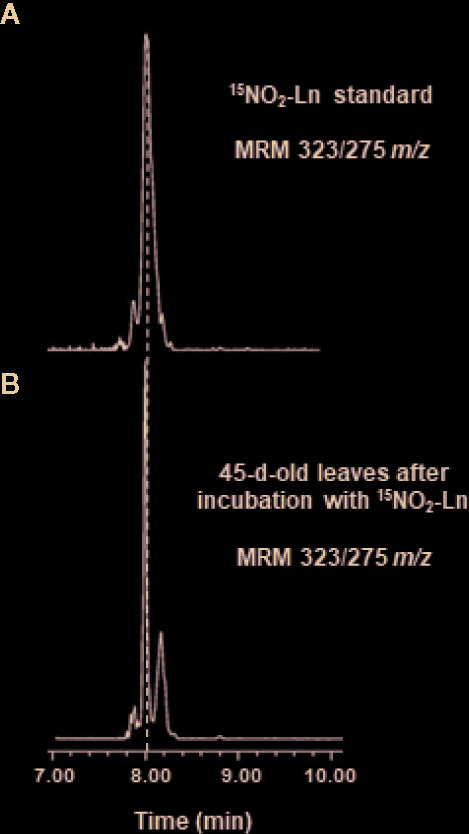
Detection of ^15^NO_2_-Ln in *Arabidopsis* leaves. 45-day-old *Arabidopsis* plants were incubated with 1 mM ^15^NO_2_-Ln for 3 h and the lipid fraction from leaves was analyzed by LC-MS/MS as it is indicated in *Materials and Methods*. **(A)** ^15^NO_2_-Ln standard with MRM transition of 323/275 *m/z*. **(B)** A chromatographic peak sharing the same retention time and *m/z* than ^15^NO_2_-Ln standard in leaves from 45-day-old *Arabidopsis* plants incubated with ^15^NO_2_-Ln. Peaks refer to a total ion intensity of 2.19 e4. Vertical dashed lines indicate peaks with the same retention time. MRM indicates multiple monitoring reaction. *m/z* indicates mass-to-charge ratio.

The capacity of NO_2_-FAs to move through the plant was analyzed. For this, ^15^N-labeled NO_2_-Ln was synthesized in order to show the presence of this NO_2_-FA in the leaves and distinguish it from the endogenous NO_2_-Ln. Thus, 1 mM ^15^NO_2_-Ln was applied to the root system of 45-day-old *Arabidopsis* plants, as indicated in *Materials and Methods*. Then, the lipid fraction obtained from *Arabidopsis* leaves was studied by LC-MS/MS **(** **Figure 2** **)**. The results showed a chromatographic peak with the MRM transition of 323/275 *m/z* **(** **Figure 2B** **)** sharing the same retention time as the ^15^NO_2_-Ln standard **(** **Figure 2A** **)** and thus highlighting the mobilization of NO_2_-FAs from the roots to the leaves of *Arabidopsis* plants.

### Characterization of GSNO Synthesis From NO_2_-Ln in *Arabidopsis* Leaves

**Figure 3:**
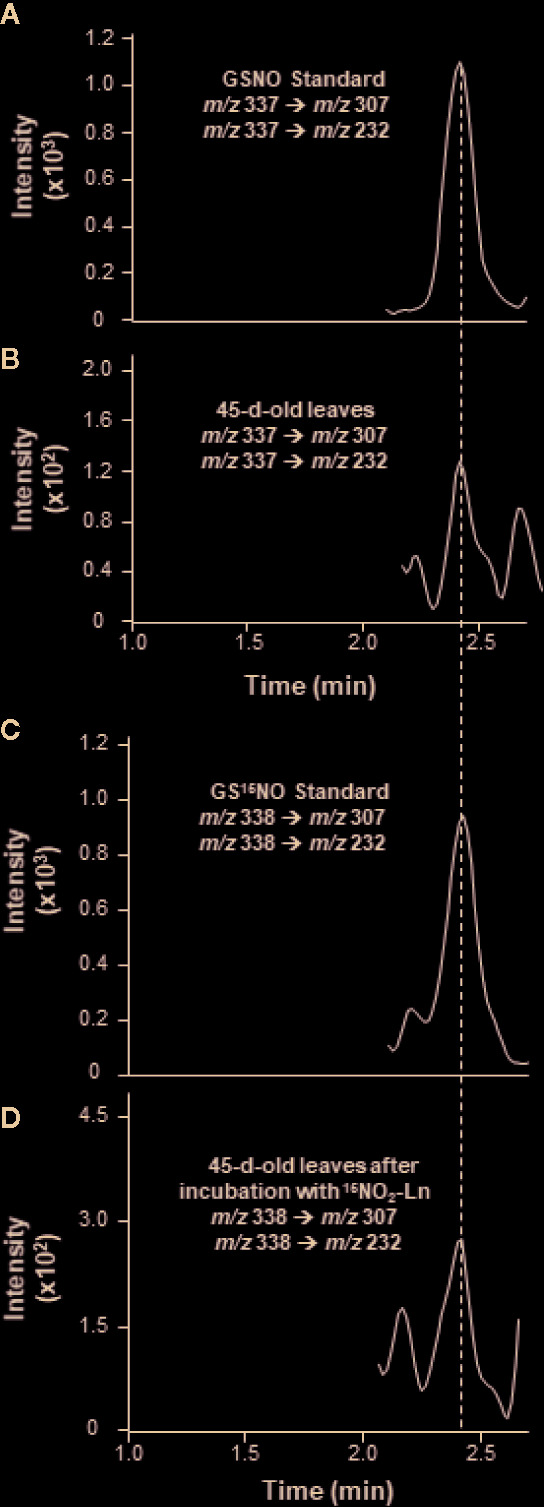
Detection of endogenous GSNO and GS^15^NO from ^15^NO_2_-Ln in *Arabidopsis* leaves by LC-MS/MS in positive ion mode. **(A, B)** The detection of endogenous GSNO in leaves from 45-day-old *Arabidopsis* plants. **(C, D)** GS^15^NO generated after the incubation of 45-day-old *Arabidopsis* plants with 1 mM ^15^NO_2_-Ln for 3 h as described in *Materials and Methods*. Peaks refer to total ion intensity. Vertical dashed lines indicate peaks with the same retention time. *m/z* is mass-to-charge ratio.

The endogenous occurrence of GSNO was analyzed by LC-MS/MS in 45-day-old *Arabidopsis* plants. The MRM scan mode was used to display the presence of a peak with transitions of *m/z* 337/307 and 337/232 specific for the fragmentation of GSNO molecule and sharing the same retention time as GSNO standard **(** **Figures 3A, B** **)**. According to the indications described in *Materials and Methods*—regarding the quantification of endogenous GSNO content, the concentration of this SNO in *Arabidopsis* leaves was 0.91 ± 0.23 pmol/mg protein (**Table 1**). These findings are consistent with previous data reported for low-mass SNO levels in *Arabidopsis* leaves (Feechan et al., 2005).

**Table 1:** Content of S-nitrosoglutathione (GSNO) in 45-d-old Arabidopsis leaves analysed by LC-MS/MS.

	pmol/mg prot	fmol/g FW
Endogenous GSNO in Arabidopsis leaves	0.91 ± 0.23	0.0018 ± 0.00045
GS^15^NO generated in leaves from Arabidopsis plants incubated with NO2−15Ln	0.50 ± 0.15	0.0011 ± 0.00033

It is important to note that the presence of GS^15^NO was assessed by LC-MS/MS in *Arabidopsis* leaves after the plant roots were incubated with ^15^NO_2_-Ln. This analysis showed a chromatographic peak sharing the same retention time as GS^15^NO standard with *m/z* 338/307 and 338/232 **(** **Figures 3C, D** **)** and thus confirming the observed peak corresponding to this low-molecular-weight SNO. Regarding the concentration of GS^15^NO detected in *Arabidopsis* leaves after the application of ^15^NO_2_-Ln was 0.50 ± 0.15 pmol/mg protein (**Table 1**). These results confirm the potential of ^15^NO_2_-Ln to generate GSNO in a significant amount, and the ability of ^15^NO_2_-Ln to travel throughout the plant system.

### NO_2_-Ln Modulates the Endogenous Levels of GSNO

**Figure 4:**
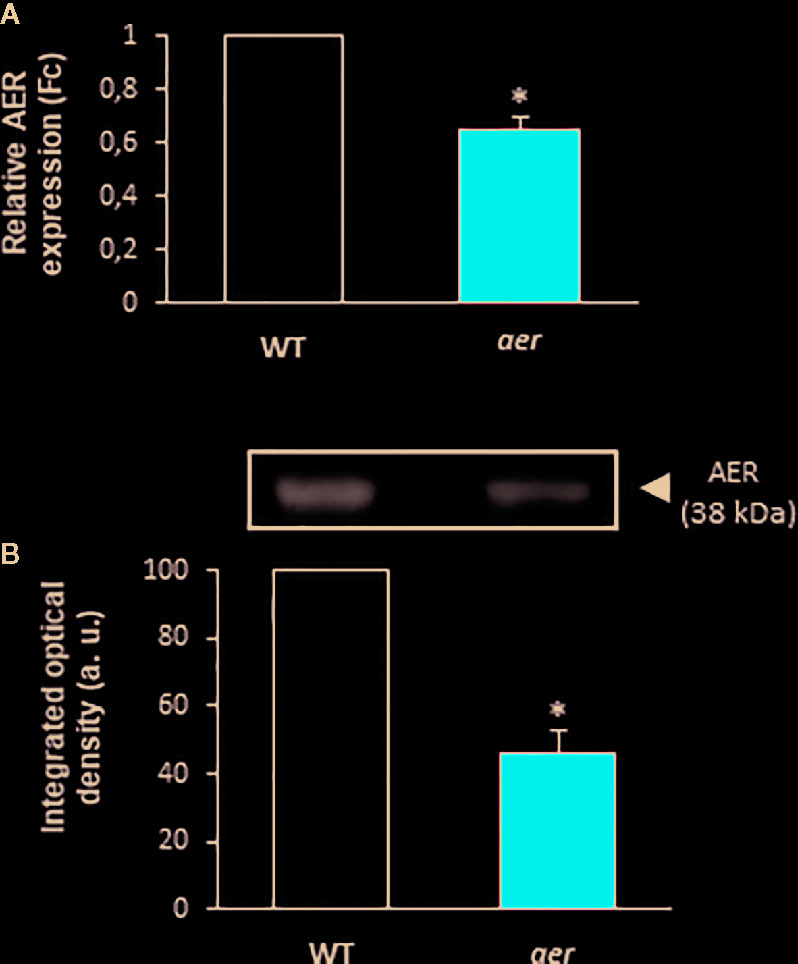
Characterization of *aer* mutant seedlings. **(A)** Gene expression in *aer* mutant plants analyzed by real-time quantitative PCR. Actine was used as internal control. **(B)** The effect of mutation on AER protein content. Twenty microgram of wild and *aer* mutant seedling were subjected to SDS-PAGE. Proteins were electroblotted onto PVDF membranes and then incubated with an antibody against AER (1:1,000). The densitometry of the bands is expressed in arbitrary units (a.u.) of integrated optical density. Data are expressed as the mean ± SEM from at least three independent samples. Differences from control values were significant at p < 0.05 (*).

**Figure 5:**
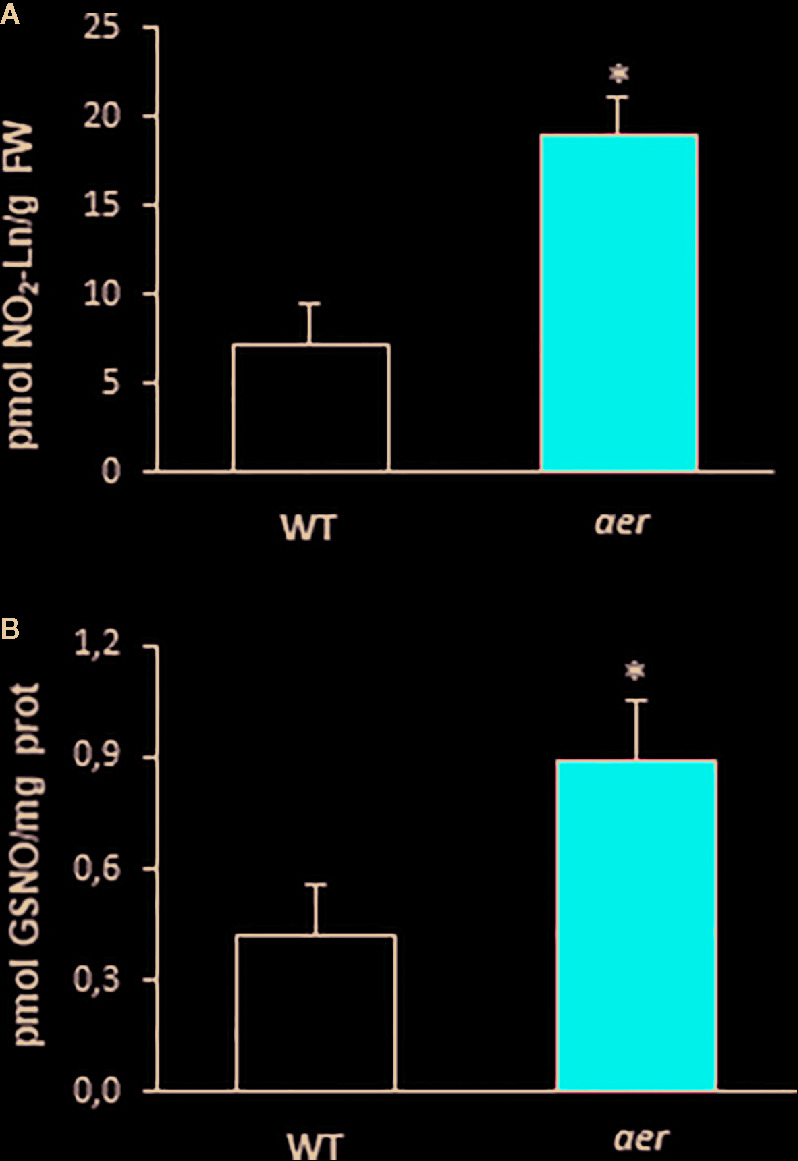
Endogenous content of NO_2_-Ln and GSNO of *aer* mutant seedlings. **(A)** The detection of endogenous NO_2_-Ln levels in both wild type and *aer* mutant 7-day-old seedlings. Lipid extracts from 7-day-old seedlings were obtained as is indicated in *Materials and Methods* and analyzed by LC-MS/MS. **(B)** The levels of endogenous S-nitrosoglutathione (GSNO) in both wild type and *aer* mutant 7-day-old seedlings detected by mass spectrometry techniques. Data are expressed as the mean ± SEM from at least three independent samples. Differences from control values were significant at p < 0.05 (*).

With the aim of support the previous demonstration about the relation between NO_2_-FAs and GSNO in plants, *Arabidopsis* Alkenal Reductase (AER, ATG16970) deficient mutant lines were used. AER enzymatic activity modulates the unsaturated fatty acid levels since it is able to reduce unsaturated to saturated bonds. Therefore, this enzyme could have the potential to modulate NO_2_-FA levels. To confirm this presumption, we used homozygous *aer* transgenic seedlings (SALK-005324C) and both transcript and protein levels were analyzed. Results show a decrease of about 35% on AER-transcript level (**Figure 4A**) and a concomitant reduction of approximately 60% in the AER-protein content (**Figure 4B**) compared to WT plants hence confirming the AER deficiency on these transgenic plants. To probe the connection between NO_2_-FAs and GSNO content, we first observed that *aer* seedlings showed higher levels of unsaturated fatty acids as linolenic acid (**Table 2**). Consequently, the decrease in AER expression resulted in a three-fold increase of NO_2_-Ln content (**Figure 5A**) importantly correlated to the observed two-fold increase of GSNO content (**Figure 5B**).

**Table 2:** Composition of fatty acids (expressed at mg fatty acid / kg FW) in both 7 d wild-type and *aer* mutant seedlings detected by mass spectrometry techniques (GC-MS). 0.002% of Ln in WT and 0.004 % of Ln in *aer* mutants are nitrated in the form of $NO_{2^{-}}$Ln.

Fatty acids	WT	*aer*
Oleic acid (18:1)	5.86 ± 0.27	8.92 ± 0.59*
Linoleic acid (18:2)	42.13 ± 1.99	61.18 ± 5.40*
Linolenic acid (18:3)	115.00 ± 4.90	158.30 ± 14.20*
Stearic acid (18:0)	11.21 ± 0.81	9.70 ± 0.41
Palmitic acid (16:0)	43.01 ± 2.53	40.00 ± 4.27

Data are expressed as the mean ± SEM from at least three independent samples. Differences were significant at p<0.05 (*).

## Discussion

For some time, the interest in the role and interaction of NO with biomolecules has significantly intensified. Most of the previous studies have mainly focused on the capability of NO to mediate several post-translational modifications (NO-PTM) such as the nitration and *S*-nitrosation of proteins. Nevertheless, in the last decade attention has focused on the ability of NO and NO-derived species to interact with non-saturated fatty acids yielding nitro fatty acids (NO_2_-FAs) (Schopfer et al., 2011; Mata-Pérez et al., 2017). These molecules have emerged as novel signaling mediators in animal and plant systems. In this respect, NO_2_-FAs can release NO in aqueous solutions and they are also able to mediate post-translational modifications of proteins through a mechanism called nitroalkylation (Lima et al., 2005; Schopfer et al., 2005; Gorczynski et al., 2007; Geisler and Rudolph, 2012; Mata-Pérez et al., 2016a; Aranda-Caño et al., 2019). These capacities confer to NO_2_-FAs relevant anti-inflammatory, antioxidant, and pro-survival properties in animal systems (Schopfer et al., 2011; Delmastro-Greenwood et al., 2014). In this sense, nitro-oleic (NO_2_-OA) and nitro-linoleic acids (NO_2_-LA) blunt pro-inflammatory responses *via* alkylation of the p65 subunit of NF-κB and they also reduce the expression of vascular-cell adhesion molecule (VCAM)-1 (Cui et al., 2006). Moreover, nitro-conjugated linoleic acid (NO_2_-cLA) is able to inhibit heme oxygenase 1 (HO-1), helping to resolve inflammation injuries (Bonacci et al., 2012). Beyond the well-defined properties of NO_2_-FAs in animal systems, it has recently been demonstrated that NO_2_-Ln is endogenously present in several plant species, including *Arabidopsis thaliana*, *Pisum sativum* or *Oryza sativa* (Mata-Pérez et al., 2016b; Mata-Pérez et al., 2017). An RNA-seq analysis showed that the incubation of *Arabidopsis* cell cultures with this NO_2_-FA promoted the induction of a large set of HSPs and several antioxidant systems such as ascorbate peroxidase (APX) or methionine sulfoxide reductase (MSRB) enzymes (Mata-Pérez et al., 2016b). In line with these results, a previous analysis with NO_2_-OA in human endothelial cell cultures determined that this NO_2_-FA was also able to prompt a defence response through greater expression of different HSPs (Kansanen et al., 2009), thus highlighting the beneficial responses which NO_2_-FAs are able to promote. Moreover, a significant rise in the levels of these species has been reported under stress conditions such as inflammation and cardiac ischemia in animal systems (Nadtochiy et al., 2009; Rudolph et al., 2010) or under salinity, mechanical wounding or heavy metal stresses in plants (Mata-Pérez et al., 2016b). Therefore, these novel NO-derived species are important in animal and plant physiology because of their capability to set up a defence response against unfavorable conditions. In this regard, NO_2_-Ln has been described to be able to regulate the function of APX to detoxify the H_2_O_2_ (Aranda-Caño et al., 2019).

### Generation of GSNO From NO_2_-Ln *In Vitro* and *In Vivo*

At present, it is well known that NO_2_-FAs are NO donors in the cell environment (Gorczynski et al., 2007; Mata-Pérez et al., 2016a). Although the capacity of NO_2_-FAs to release NO was firstly considered to be of minor significance *in vivo* and less than 1% *in vitro*, recent studies have shown that NO_2_-FAs can generate NO in a similar way to GSNO, that is considered the major biological NO reservoir and a key regulator of a wide range of physiological and stress-related processes in plants (Mata-Pérez et al., 2016a; Begara-Morales et al., 2018).

Therefore, NO_2_-FAs, as NO_2_-Ln, provide a significant source of NO in plants and together with the high content of GSH in living systems, it could contribute to the total pool of GSNO and SNOs in cells. Based on this background, we investigated both the *in vitro* and *in vivo* capacity of NO_2_-Ln to modulate the generation of GSNO. In this regard, using different concentrations of this NO_2_-FA, the incubation of NO_2_-Ln with GSH was analyzed by mass spectrometry and the *in vitro* formation of GSNO was noted. The formation of GSNO was concentration-dependent, displaying the higher levels of this SNO after incubation of 1 mM of NO_2_-Ln with 1 mM GSH. Furthermore, to confirm whether NO_2_-Ln can modulate the levels of GSNO *in vivo*, we firstly studied the mobilization of ^15^NO_2_-FAs through the plant and, to achieve it, we undertook the synthesis of ^15^N-labeled NO_2_-Ln (^15^NO_2_-Ln). This labeled-NO_2_-FA was used to differentiate its action from endogenous NO_2_-Ln in *Arabidopsis* leaves (Mata-Pérez et al., 2016b; Mata-Pérez et al., 2017). In this sense, ^15^NO_2_-Ln was applied to the root system and its occurrence was analyzed in the leaves of 45-day-old *Arabidopsis* plants. By using high-accuracy mass spectrometry approaches, we detected the presence of ^15^NO_2_-Ln in leaves from plants pre-incubated with this labeled NO_2_-FA. In line with these results, prior studies have shown the application of the fatty acid heptadecanoic acid (17:0) to the root system of several plant species including *Glycine max*, *Zea mays* or *Lycopersicum esculentum*, leading to its detection in leaves and, after the application in leaves, it was detected in both leaves higher on the plant and in roots. These results indicate translocation and the authors conclude that it could probably take place by the phloem (Terzaghi, 1989). Therefore, it has been shown that fatty acids or NO_2_-FAs can travel and exert their signaling actions throughout the whole plant.

After having shown that NO_2_-Ln can be mobilized across the plant and reach the shoots, the capability of ^15^NO_2_-Ln to modulate the levels of ^15^N-labeled GS^15^NO was studied. Firstly, using a LC-MS/MS approach similar to that described elsewhere (Tsikas et al., 2013) with some modifications, we assessed the endogenous occurrence of GSNO in *Arabidopsis* leaves. The endogenous GSNO level of 7-day-old plants **(** **Figure 5B** **)** detected is lower than that detected in 45 old-day plants (**Table 1**). Based on these results, GSNO levels appear to decrease during *Arabidopsis* development.

The endogenous GSNO content detected was consistent with levels previously reported by Feechan et al. (2005) in *Arabidopsis* after using a 5-kDa cut-off membrane in *Arabidopsis* leaf extracts and therefore detecting all low molecular weight SNOs. Nevertheless, this endogenous GSNO content is significantly lower than the nanomolar concentration described by Airaki et al. (2011) in *Arabidopsis* leaves. This apparent discrepancy could be a consequence of the acidic media used for protein extraction in Airaki et al. (2011). In that work, GSNO data at the nanomolar level are likely to be overestimated by an artifactual production of GSNO under these acidic extraction conditions when both nitrite and GSH are present (Broniowska et al., 2013). In addition, in the present work the GSNO detection was performed by LC-MS/MS that is a more sensitive technology that LC-MS to detect endogenous GSNO levels (Tsikas and Hanff, 2018). After showing that GSNO was endogenously present in *Arabidopsis* leaves, we incubated plants with ^15^NO_2_-Ln in the same way as previously described and we studied the occurrence of GS^15^NO. The results displayed the presence of GS^15^NO in the shoots after the treatment, thus confirming that NO_2_-Ln can modulate the generation of GSNO *in vivo*. We incubated plant roots with 1mM of ^15^NO_2_-Ln and a concentration of GS^15^NO of 0.50 ± 0.15 pmol/mg was quantified in leaves. Different reasons emerge to explain this apparent low GS^15^NO detection. It was previously described that the use of 1mM NO_2_-Ln can release NO *in vitro* in a ratio of 0.21 µM/min (Mata-Pérez et al., 2016a) or 12.6 µM/h. Therefore, during plant treatment with 1mM ^15^NO_2_-Ln, the ^15^NO generated will be in the µM range. Consequently, the *in vivo* detection of ^15^NO_2_-Ln-dependent generation of GS^15^NO will be apparently low compared with the initial concentration of the labeled nitro fatty acid. In addition, NO_2_-FAs are more abundant esterified in complex lipids than in the free form (Fazzari et al., 2019). In this line, oral administration of dogs with NO_2_-OA confirmed that the main distribution of this NO_2_-FA is esterified in different complex lipids, especially triacylglycerides (TAGs) (Fazzari et al., 2019). Thus, a similar situation could be happening in *Arabidopsis* plants incubated with NO_2_-Ln, in which a substantial percentage of the initial amount of NO_2_-Ln could be esterified in complex lipids, therefore not being able to release NO and, ultimately, generate GSNO. In this regard, the total free NO_2_-Ln detected in *Arabidopsis* seedlings is around 4 pmol/g FW in control plants being increased about two-fold after different abiotic stresses (Mata-Pérez et al., 2016b). In addition, NO_2_-FAs are electrophile molecules that can mediate post-translational modification of proteins by nitroalkylation (Aranda-Caño et al., 2019) and therefore not all pool of NO_2_-Ln would not contribute to *in vivo* GSNO generation. Consequently, the GSNO concentration observed is consistent with the NO_2_-Ln capacity to release NO and its endogenous abundance.

**Figure 6:**
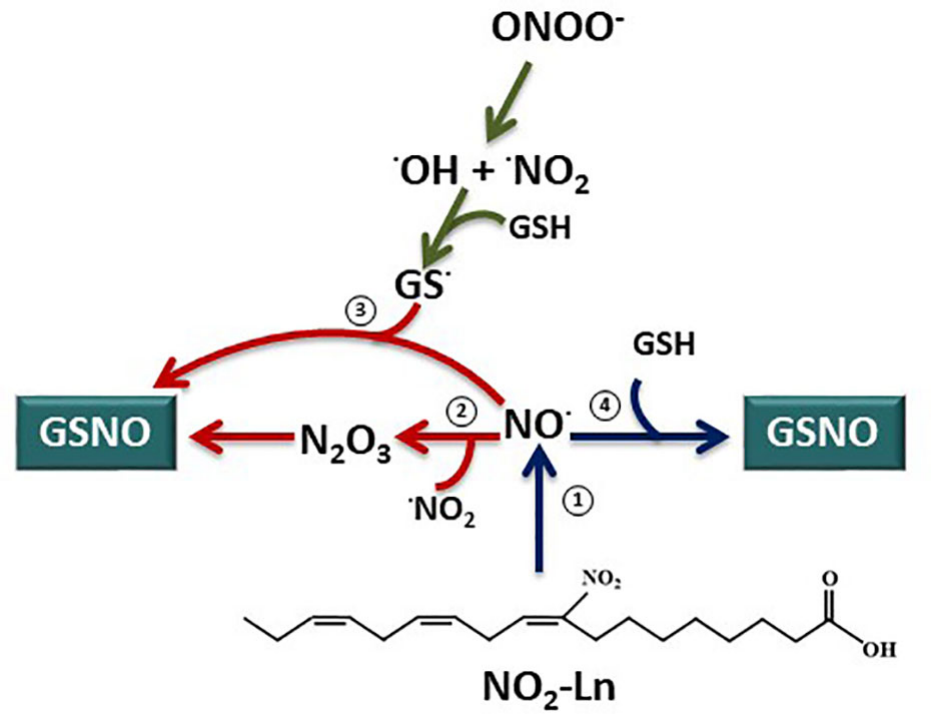
GSNO biosynthesis from nitrolinolenic acid. Nitrolinolenic acid (NO_2_-Ln) has the capacity to release nitric oxide (NO) at a physiological pH and temperature (1) (Mata-Pérez et al., 2016a). Therefore, this NO can contribute to the intracellular levels of S-nitrosoglutathione (GSNO). However, the exact mechanisms for GSNO generation remains to be elucidated. Instead of a direct reaction of NO and GSH, the formation of N_2_O_3_ as an intermediary (2) or the reaction of NO with the glutathionyl radical (GS.) (3) have been proposed as pathways for GSNO synthesis. Interestingly, the direct interaction of NO and GSH leading to GSNO formation appears to be possible at a submicromolar concentrations of NO (Kolesnik et al., 2013). Consequently, keeping in mind the submicromolar generation of NO from the NO_2_-Ln (0.21 µM/min) (Mata-Pérez et al., 2016a), the NO_2_-Ln-dependent formation of GSNO will take place by a direct reaction of NO released from the nitro fatty acid and GSH (4).

It is worth noting that the exact mechanisms leading to GSNO formation remains unclear (Broniowska et al., 2013; Zaffagnini et al., 2016; Begara-Morales et al., 2018). Instead of a direct reaction of NO with GSH to generate GSNO, the most probably pathways to generate GSNO are the interaction of NO with the glutathionyl radical (GS.) or the formation of N_2_O_3_ as an intermediary **(** **Figure 6** **)** (Broniowska and Hogg, 2012; Broniowska et al., 2013; Kolesnik et al., 2013; Begara-Morales et al., 2018). However, it is possible the direct nitrosation of GSH by NO leading to GSNO at submicromolar concentrations of NO (Kolesnik et al., 2013). In this line, NO_2_-Ln is able to release NO at a rate of 0.21 uM/min at a physiological pH (Mata-Pérez et al., 2016a) and therefore it generates NO at a submicromolar levels. Consequently, the NO_2_-Ln-dependent generation of GSNO described in this work could be performed as a direct interaction of NO released from NO_2_-Ln and GSH **(** **Figure 6** **)**.

### The Modulation of Cellular Levels of NO_2_-Ln Directly Influences the GSNO Production

In order to clarify if the capacity of NO_2_-Ln to modulate GSNO levels could have physiological implications, we used mutant plants that are able to regulate endogenous levels of NO_2_-Ln. In this work, we have demonstrated the capacity of alkenal reductase (AER) enzyme to modulate endogenous levels of NO_2_-FAs, concretely NO_2_-Ln, as previously reported for its homologous in human and rats (prostaglandin reductase, PTGR-1) (Vitturi et al., 2013). A decrease of AER gene expression and protein content exhibited a three-fold increase of NO_2_-Ln and two-fold in GSNO levels, confirming the capacity of NO_2_-Ln to control the abundance of endogenous GSNO and therefore the NO-dependent signaling in plants (Begara-Morales et al., 2018). Furthermore, it is important to note that GS^15^NO-detected levels represent approximately 50% of those detected in the control situation, which taking into account the multiple possible targets of NO_2_-Ln, should be considered as a very important contribution. Regarding the mobility of NO and NO-derived molecules, GSNO have been detected in vascular bundles and epidermal cells of several plant species (Chaki et al., 2011a; Chaki et al., 2011b). Hence, it has been postulated that the phloem seems to be an active site for NO metabolism and also for GSNO generation (Gaupels et al., 2017). All these results may suggest that the phloem can act as a key tissue location for the metabolism of NO_2_-FAs, NO and consequently GSNO.

On the other hand, SNOs and GSNO can mediate NO-PTMs like *S*-nitrosation of different protein targets hence triggering notorious consequences in their enzymatic activities or their protein functions. In this respect, GSNO and SNOs have been identified in numerous plant situations, highlighting the involvement of these molecules in diverse stressful situations. It is well documented that *S*-nitrosation plays a key role in plant immunity (Feechan et al., 2005; Romero-Puertas et al., 2008; Tada et al., 2008). For instance, knockout plants for the GSNOR1 enzyme (*gsnor1*) showed a high content of GSNO and indirectly of SNOs related to more disease susceptibility compared to WT plants (Feechan et al., 2005). Furthermore and regarding abiotic-stress conditions, a modulation in GSNO and SNO levels has also been reported in different plant species (Valderrama et al., 2007; Chaki et al., 2011a; Chaki et al., 2011b), supporting the contention that the abiotic-stress response can be mediated, at least in part, by *S*-nitrosation-signaling of key protein targets such as pea APX, which upregulates its activity during salt stress (Begara-Morales et al., 2014a).

Related to what it has previously mentioned, NO_2_-FAs are lowly abundant in their free form (Tsikas et al., 2009; Mata-Pérez et al., 2016b). Actually, most of these nitro-derivatives are thought to be protein-adducted or putatively esterified with complex lipids being part of cell membranes (Rubbo, 2013). Certain conditions such as the nitro-oxidative burst taking place under several stress circumstances (for instance salt, heavy metal or wounding stresses in *Arabidopsis* (Mata-Pérez et al., 2016b), can prompt the release of free NO_2_-FAs from the pool of adducted proteins (Padilla et al., 2017) or certain other signals could be de-esterifying complex lipids from cell membranes with the subsequent liberation of free NO_2_-FAs. Bearing this in mind, we would like to highlight that a concomitant increase on the free-NO_2_-FA pool together with the high abundance of GSH in living systems creates a perfect environment for the direct modulation of cellular levels of GSNO. This direct relation may have relevant consequences in plant physiology and it may facilitate the understanding about the modulation and control of the SNO-signaling pathway in plants.

**Figure 7:**
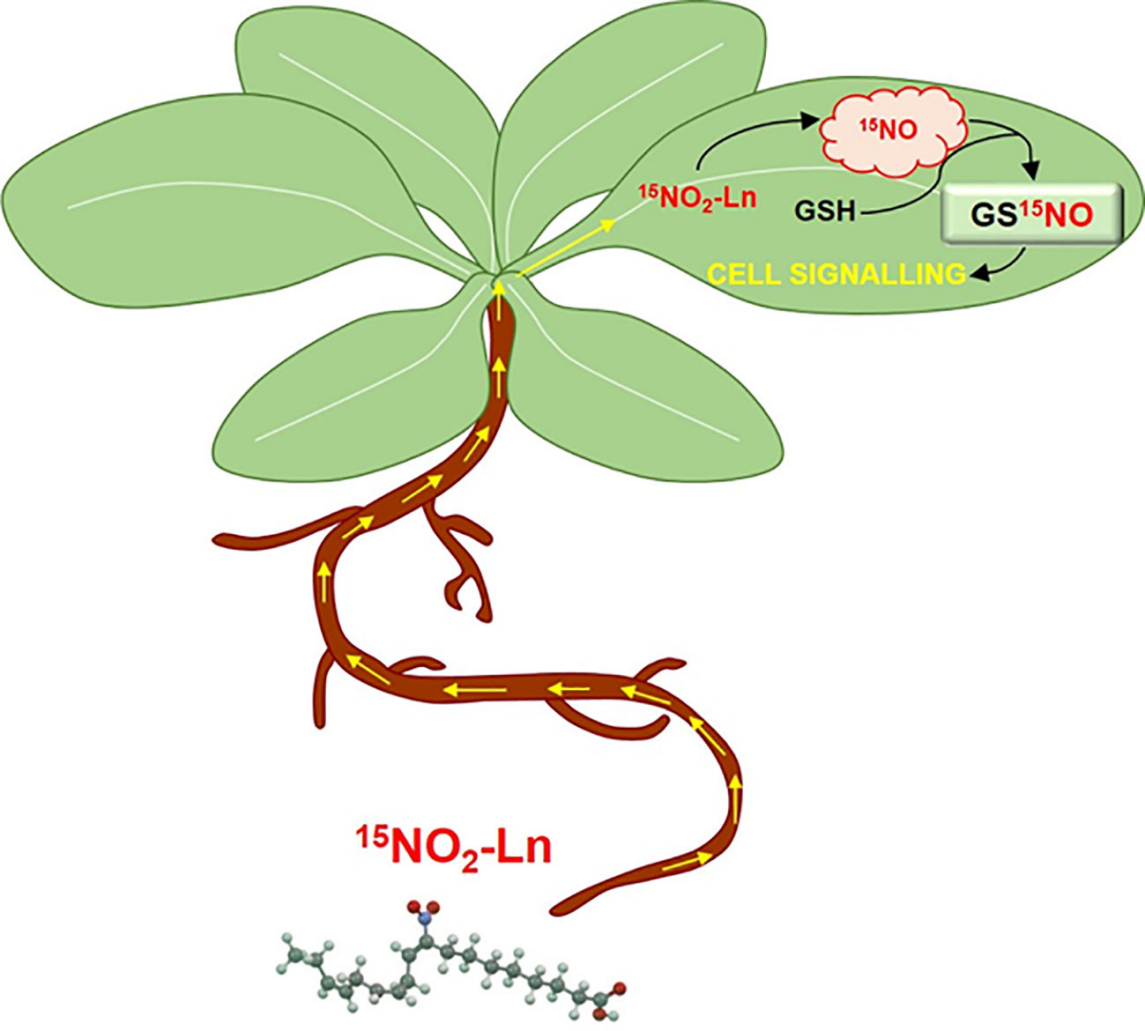
Modulation of GSNO-signalling pathway by NO_2_-Ln in *Arabidopsis* leaves. NO_2_-Ln is up-taken by the root system and transported to plant leaves. Once in these organs, NO_2_-Ln can release nitric oxide (NO) and mediate the S-nitrosation of abundant glutathione (GSH) present in plant leaves and leading to the formation of S-nitrosoglutathione (GSNO). The generation of this low-molecular weight S-nitrosothiol (SNO) from NO_2_-Ln can affect the SNO-signaling pathway by modulating the transport and storage of NO, the response to several (a)biotic stress conditions or mediating the ability of SNO to perform post-translational modifications.

Finally, this behavior can be exemplified in the proposed model for the modulation of GSNO-signaling pathway by NO2-Ln (**Figure 7**). NO2-FAs have recently been shown to be present in several organs and organelles of diverse plant species (Mata-Pérez et al., 2016b; Mata-Pérez et al., 2017). In fact, these molecules can be mobilized from their cell locations through the plant organs and reach the shoots. This together with the fact that NO2-FAs have been described as physiological NO donors and the high abundance of the antioxidant GSH in living systems, may establish a proper cell environment for the formation of GSNO. The generation of this low-molecular-weight SNO from NO2-FAs can affect the SNO-signaling pathway by modulating the transport and storage of NO, the response to several (a)biotic stress conditions, or the mediating ability of SNOs to perform NO-PTMs. Thus, NO2-FAs can be considered new key modulators in the GSNO-dependent signaling cell response during physiological and stress conditions in plants.

## Conclusions

Our study provides further relevant insights into the signaling mediated by NO_2_-FAs in plants. Data presented in this study provide novel information concerning the GSNO biosynthesis mechanisms, indicating that modulation of cellular levels of NO_2_-FAs can directly influence the GSNO levels. In fact, the key property of NO_2_-Ln to release NO allows to act as a powerful signaling molecule since it is able to induce functional changes mediated by NO or NO-related molecules including post-translational modifications such as *S*-nitrosation. Therefore, NO_2_-FAs can be considered key players regulating the NO-bioactivity, so that the study of the interactions between NO_2_-FAs and GSNO will increase the knowledge about SNO-signaling pathway in plants. On the basis of these results, the control of GSNO by NO_2_-FAs has emerged as an interesting regulation point of SNO-bioactivity in plant physiology and during (a)biotic stress processes.

## Data Availability Statement

The original contributions presented in the study are included in the article/**Supplementary Material**. Further inquiries can be directed to the corresponding author.

## Author Contributions 

This work was conceptualized by JB. Experiments were performed by all authors. The data were analyzed by CM-P, MP, JB-M, and JB. The paper was written by CM-P, MP, JB-M, RV, and JB.

## Conflict of Interest

The authors declare that the research was conducted in the absence of any commercial or financial relationships that could be construed as a potential conflict of interest.
