# Supplementary figures and images for: Endogenous Biosynthesis of S-Nitrosoglutathione From Nitro-Fatty Acids in Plants
Source: Front Plant Sci. 2020 Jun 30;11:962. doi: 10.3389/fpls.2020.00962 (PMC7340149; doi:10.3389/fpls.2020.00962)

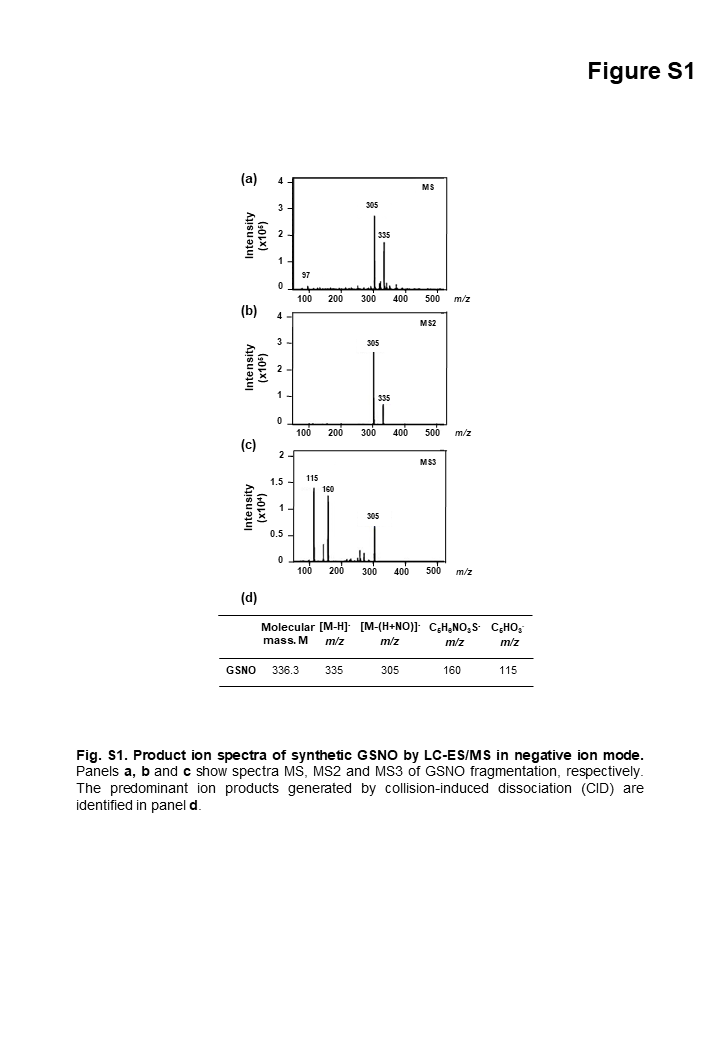

Supplement: Supplementary file 1 [file Image_1.tif]

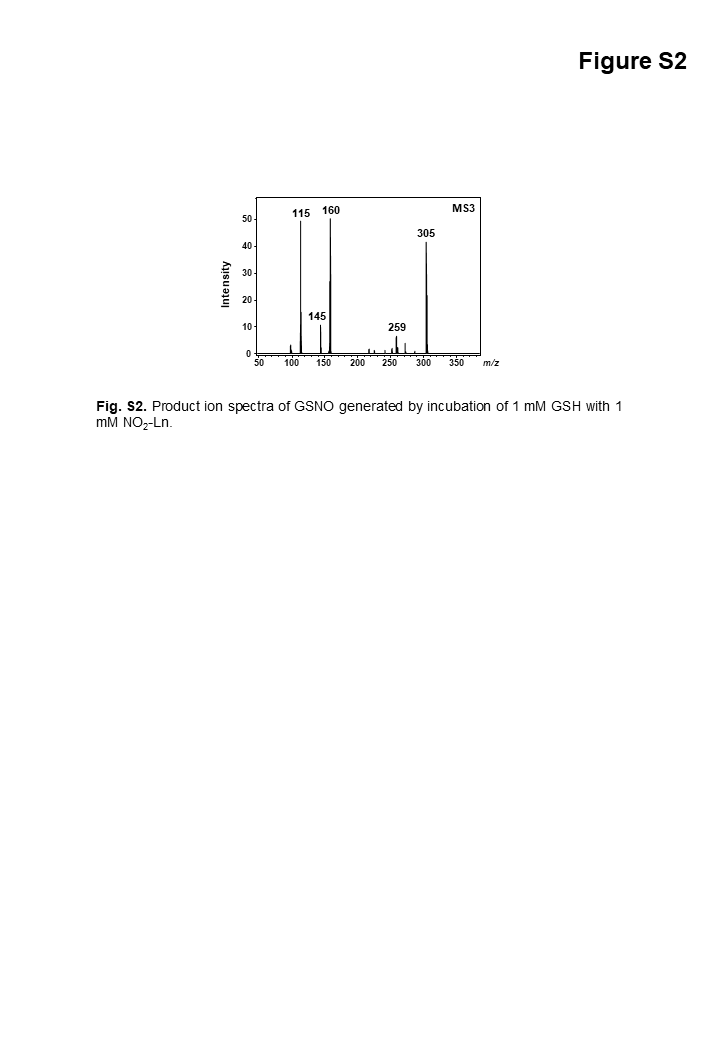

Supplement: Supplementary file 2 [file Image_2.tif]

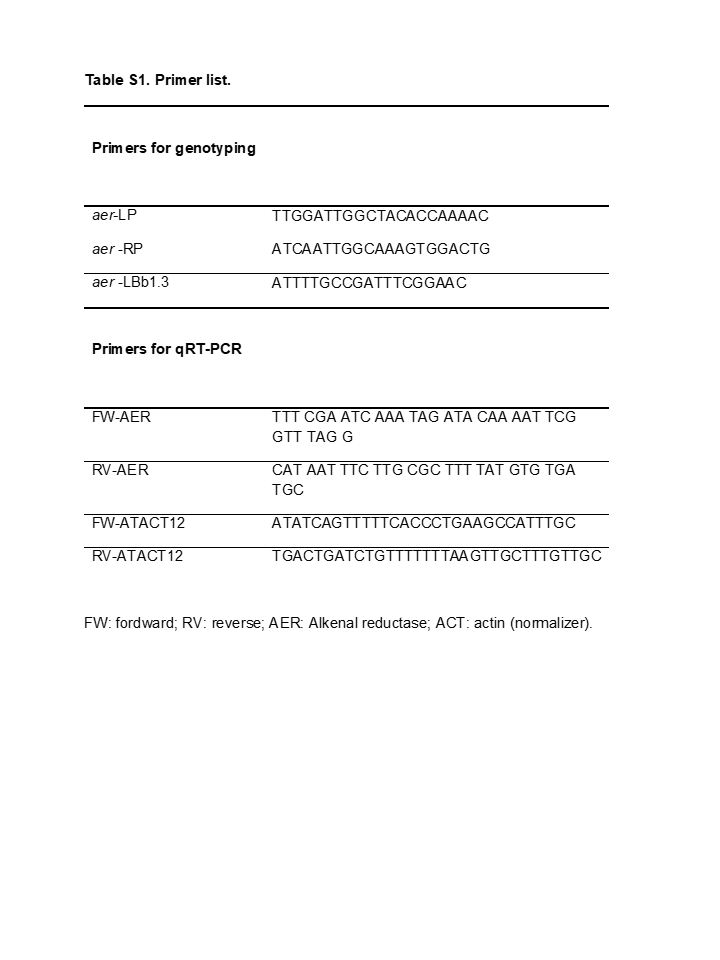

Supplement: Supplementary file 3 [file Image_3.tif]

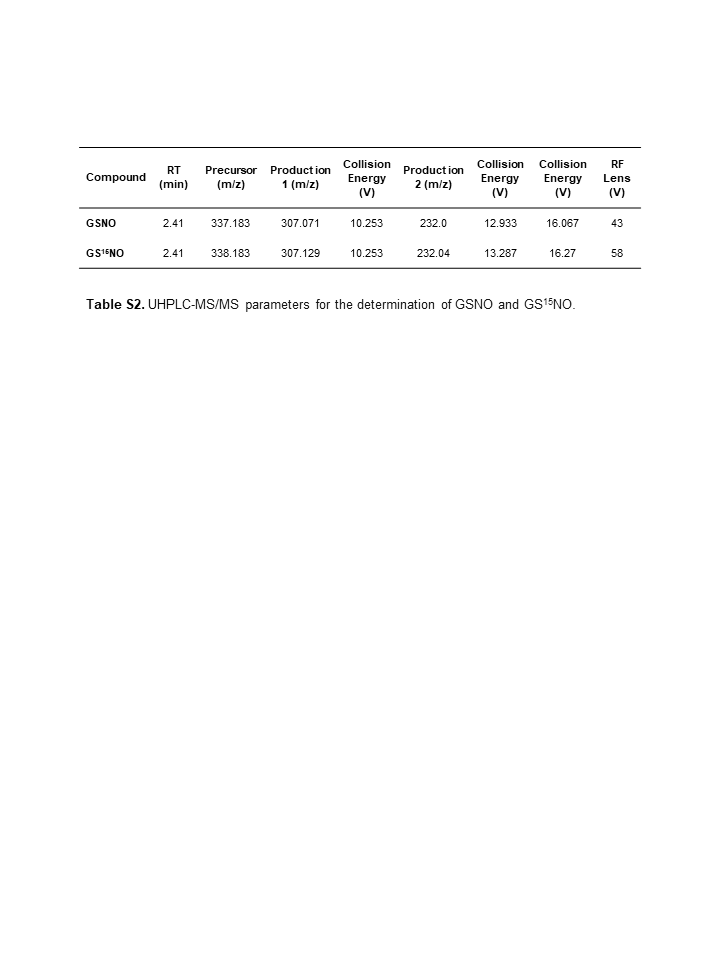

Supplement: Supplementary file 4 [file Image_4.tif]
